# Differential expression of Fas receptors (CD95) and Fas ligands (CD95L) in HIV infected and exposed uninfected children in Cameroon versus unexposed children

**DOI:** 10.11604/pamj.2019.34.39.15038

**Published:** 2019-09-18

**Authors:** Béatrice Dambaya, Céline Nguefeu Nkenfou, Georgia Ambada, George Mondinde Ikomey, Linda Mekue Mouafo, Nicole Ngoufack, Elvis Ndukong Ndzi, Georges Této, Aubin Nanfack, Nelson Sonela, Joseph Fokam, Njiokou Flobert, Vittorio Colizzi, Alexis Ndjolo

**Affiliations:** 1 Chantal Biya International Reference Centre for Research on HIV/AIDS prevention and management-CBIRC, Yaounde, Cameroon; 2 Faculty of Sciences, University of Yaounde I, Yaounde, Cameroon; 3 Higher Teachers’ Training College, University of Yaounde I, Yaounde, Cameroon; 4 Faculty of Medicine and Biomedical Sciences, University of Yaounde I, Yaounde, Cameroon; 5 Faculty of Sciences, University of Dschang, Dschang, Cameroon; 6 University of Tor Vergata, Rome, Italy

**Keywords:** HIV, infected children, exposed non-infected children, FasL, FasR, expression level

## Abstract

**Introduction:**

The number of HIV exposed uninfected (HEU) infants is increasing as vertical transmission is reducing. This subpopulation requires more investigations. This study aimed at comparing the expression level of soluble Fas receptors (FasR) and ligands (FasL) between HIV infected, HEU and unexposed children.

**Methods:**

Eighty eight HIV-1infected, 86 HEU and 38 HIV unexposed children were recruited. Soluble FasR and FasL were measured in their plasma. Mann-Whitney U-Test was used to compare groups with 95% confidence. Spearman coefficient was used to test the correlation with CD4 and viral load (VL).

**Results:**

Overall plasma levels of FasR were higher than that of FasL. The concentration of FasR and FasL were significantly higher in HIV-1 infected children in comparison to HEU and unexposed children. There was no difference in the plasma level of FasL in HIV infected compared to HEU children. A significant difference was observed between HIV infected children and HEU children (P=0.001) for the FasL. FasR were higher in both HIV infected and unexposed children compared to HEU children. There was a positive correlation (rs=+0.4; p=0.01) in ARV treated children between CD4 count and FasL concentration. Significant negative correlation (rs=-0.3; p=0.040) in ARV naïve children was observed between CD4 percentage and FasL. Significant and positive correlation (rs=+0.4; p=0.008) was observed between the VL and FasL in HIV infected, treated or not.

**Conclusion:**

HEU children differ from HIV infected and unexposed children as the level of FasL/R expression is concerned. HEU should be considered different from HIV unexposed although exempt from virus as some immune dysfunctions have been reported among them.

## Introduction

The AIDS epidemic has harshly affected child mortality all over the world but intervention to prevent mother to child transmission has successfully reduced the risk of HIV transmission to < 1% in resource rich settings [1]. In sub-Saharan African regions, people living with HIV/Aids account for 70% worldwide [2]. Most of children living in this part of the world are infected via their HIV-positive mothers during pregnancy, childbirth or breastfeeding [2]. Some of them remained asymptomatic for several years despite the absence of therapy [3], unfortunately 50% of them die before their second anniversary [4]. Despite the greatest success of ART in HIV infection history, prevention of mother to child transmission remains an important challenge especially in sub-Saharan African countries where HIV-exposed uninfected (HEU) infants are more susceptible to morbidity and mortality due to many infectious mechanisms than their unexposed peers [5]. HEU infants represent a growing population in pediatric, they can represent nearly 30% of the newborn population in some endemic nations [6]. Even if these infants are not infected, they are affected by the virus and by the treatment receive by their mother during pregnancy to prevent transmission [7]. Infection caused by the Human immunodeficiency virus type 1 (HIV-1) is characterized by a progressive and severe depletion of CD4+ T lymphocytes [8]. This phenomenon is observed in both adult and children. In adults, T cell immune activation appears to be higher in sub-Saharan Africa than in industrialized countries [9]. The understanding of the HIV-1 disease pathogenesis requires an appreciation of mechanisms involved in the loss of CD4+ T cells. Apoptosis, one type of a programmed cell death, plays an important role in the mechanisms of HIV/AIDS infection. One apoptotic pathway is the Fas mediated mechanism during activation induced cell death (AICD) system, a key regulator of the process in normal and malignant T cells [10, 11]. Previous studies carried out on adults in Cameroon, a country characterized by a high genetic variability of HIV virus have shown the increase in plasma levels of Fas receptors (FasR) and Fas ligands (FasL) in HIV-positive compared to HIV negative individuals [12]. To date, a number of studies have shown evidence of various immunological abnormalities among HEU children [13], but only few are documented on the expression level of Fas in Sub Saharan African pediatric context particularly in HIV-exposed uninfected children. This study aimed firstly, to evaluate the level of soluble Fas receptors and ligands in HIV-infected children vertically infected, receiving antiretroviral treatment (ART) or not, and HEU children and unexposed children (controls); secondly, to assess the correlation between plasma concentrations of Fas receptors/ligands, CD4 lymphocyte cells counts and HIV-1 viral load in HIV infected children.

## Methods

**Study design:** plasma were obtained from 88 HIV-1 infected children of a study cohort of Long Term non Progressor-Elite Controller (LTNP-EC), initiated in 2012 at “Chantal Biya” International Reference Centre (CBIRC), including 48 girls and 40 boys perinatally infected with HIV. Eighty-six HIV exposed uninfected (HEU) children and 38 unexposed children as control group were also enrolled. Demographics, clinical information and the current ART regimen were obtained at the time of enrollment.

**Ethical considerations:** the ethical clearance was obtained from the National Ethics Committee under the n° 103/CNE/SE/2012. Children vertically infected with HIV attending Chantal Biya International Reference Centre (CBIRC) for their biological examinations were enrolled upon signed informed consent by their parents or guardians. Demographics and clinical data were recorded from each child using a questionnaire as well as the child's medical record. Children born to “healthy mothers-HIV negative” were also recruited with the consent of their parents as controls. These children were consulting for reasons unrelated to HIV/AIDS, such as cough, skin disease and suspected malaria.

**Blood collection:** five milliliters of blood were collected from each child included in the study. Part of whole blood was used for CD4 count and the rest was centrifuged at 1,000 g for 10 minutes, plasma was aliquoted and stored at -20^o^C for further analyses.

**Determination of CD4 counts:** CD4 cell counts were quantified using a FACScalibur flow cytometer (Becton-Dickinson, Immuno-cytometry System, San Jose, CA, USA).

**Determination of viral load:** HIV-1 viral load was determined from plasma by Abbott m2000rt Real-Time HIV-1 assay (Abbott Molecular Diagnostics, Wiesbaden, Germany), using the 200 μl protocol with a detection limit of 160 copies/ml (1.8log).

**Measurement of soluble Fas (sFas) levels:** the concentrations of soluble Fas receptor and soluble Fas ligand in plasma samples were determined using immunoenzymatic sandwich ELISA method. The kits from Quantikine^®^, R&D Systems, UK were used. The samples were analyzed according to the manufacturer' instructions and each sample was tested in duplicate. Optical densities were measured at 450 nm with an ELISA reader (Thermofisher Scientific type 357 Microplate Reader, USA). A standard curve was used to extrapolate the concentration of soluble Fas receptor and ligand in plasma samples.

**Statistical analysis:** data were analyzed using GraphPad prism version 6. Plasma levels of both Fas receptors and Fas ligands in HIV infected treated or naïve infants and in HIV negative, exposed uninfected and unexposed uninfected group, were compared using the Mann-Whitney U-Test. Correlation in each group of children between CD4+ cell counts and viral load was assessed using Spearman test, and a P-value of less than 0.05 was considered statistically significant.

## Results

**Figure 1:**
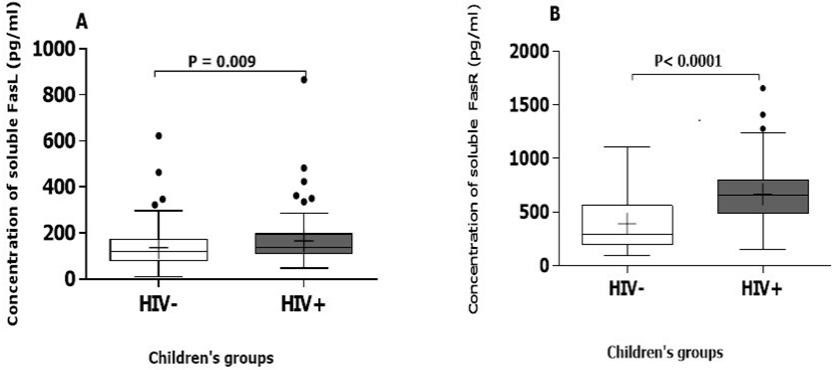
Plasma concentration of Fas ligands (FasL) (A) and Fas receptors (FasR) (B) in HIV infected and uninfected children (HEU and unexposed)

**Figure 2:**
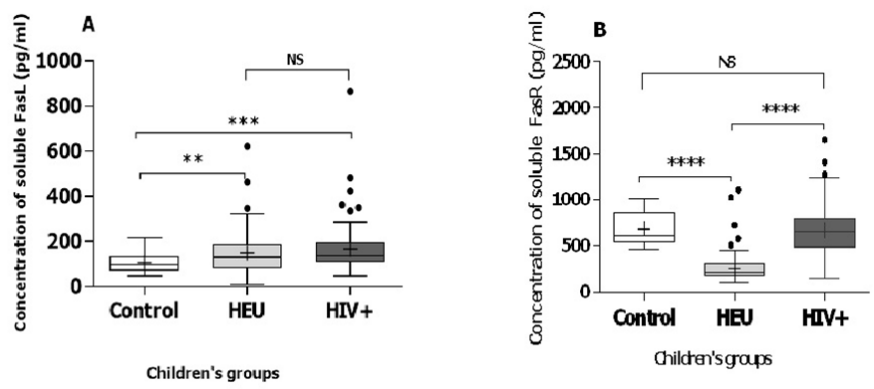
Plasma concentration of Fas ligands (A) and Fas receptors (B) in control (unexposed), HEU (Exposed Uninfected) and HIV+ (infected children). **=0.01; ***=0.001; ****=0.0001; NS: not significant

The overall population consists of 212 children aged from 9 months to 15 years with 112 girls (52.83%) and 100 boys (47.16). Our study population includes 88 HIV-1 infected children aged 1 to 15 years, mean ± SD (6.70 ± 3.23), 86 HIV exposed uninfected (HEU) children aged 9 months to 13 years, mean ± SD (5.53±2.01) and 38 healthy children as control group aged from 1 to 15 years, mean ± SD (7 ± 2.24). The description of the study population is summarized in Table 1. Plasma concentrations of Fas receptors were higher than that of Fas ligands in the overall study population. Plasma concentrations of FasL were higher in HIV-1 infected children (median=136.5pg/ml IQR=91.68 pg/ml) compared to HEU and unexposed children (median=120.6 pg/ml IQR=84.40 pg/ml) with a p value of 0.009 (Figure 1 A). Plasma levels of FasR were also higher in HIV-1 infected children (median=657.7pg/ml IQR=365.28 pg/ml) compared to HEU and unexposed children (median=289 pg/ml IQR=314.81 pg/ml) with p<0.001 (Figure 1 B). Comparing the groups two by two, HEU have a higher amount of FasL than HIV unexposed children (p=0.010). There was no statistical difference in the amount of FasL between HIV infected children and HEU (p=0.260). On the other hand, HIV infected children have a higher concentration of FasL than unexposed children (p=0.001) (Figure 2 A). For the FasR, expression level was the lowest in HEU. HEU express less FasR than HIV unexposed children (p=0.001). HIV infected children express more FasR than exposed uninfected children (p=0.001). Overall, there was no significant difference in the expression level of FasR between HIV unexposed and HIV infected children (Figure 2 B).

**Table 1:** Description of the study population

Gender	N = (212)	%
Male	100	47.20
Female	112	52.80
**Age Group** (Years)		
< 5	94	44.33
5-10	55	25.94
>10	63	29.71
**HIV infected**	**88**	**41.50**
Initiation of ART *(in months)*	44	50
At birth	5	11.36
After 1-2	8	18.18
After 3-5	8	18.18
After 6-8	6	13.63
> 8	15	34.09
Unknown	2	4.54
ART-Naïve	44	50
**HIV negative**	**124**	**58.48**
HIV exposed uninfected (HEU)	86	69.35
HIV unexposed	38	30.64

**Figure 3:**
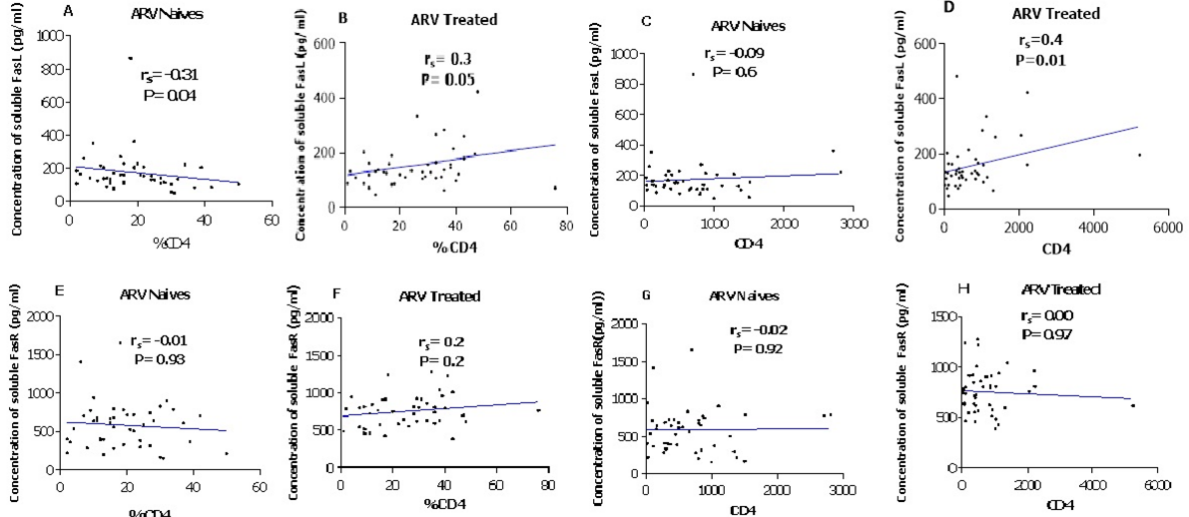
Correlation between CD4+ cell count (percentage and absolute value) and plasma concentration of Fas ligands (A (ARV naives, %CD4), B (ARV treated, %CD4), C (ARV naives, absolute CD4), D (ARV treated, absolute CD4)) and Fas receptors (E, (ARV naives, %CD4), F (ARV treated, %CD4), G (ARV naives, absolute CD4), H (ARV treated, absolute CD4)) in 44 ARV naives and 44 ARV treated children

**Figure 4:**
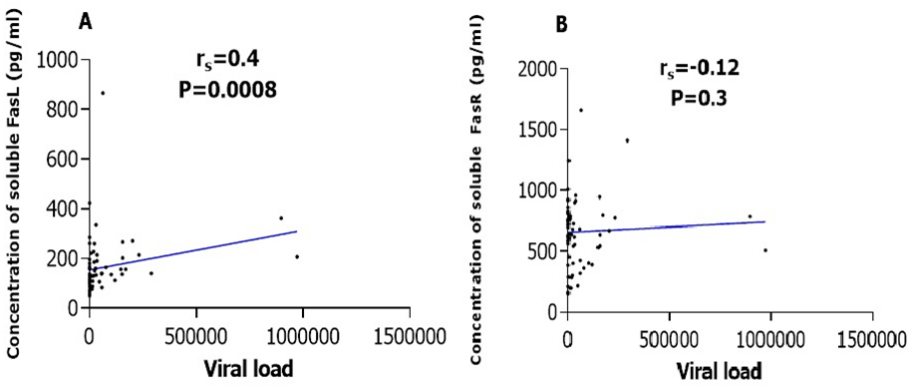
Correlation between viral load and plasma level of FasL (A) and FasR (B) in HIV infected children

Among the 44 HIV infected, ART naïve children we found a negative correlation between the percentage of CD4 and the expression level of Fas ligands (r_s_=-0.31; p=0.040) and receptors (r_s_=-0.01; p=0.93). In the group of the 44 HIV infected and ARV treated children, a positive correlation between both ligands and receptors with CD4, a significant correlation between the levels of CD4 absolute value and ligands (r_s_=0.4; p=0.010) and a weak correlation with CD4 percentage (r_s_=0.3; p=0.050), (r_s_=0.2; p=0.200) and FasL were observed (Figure 3). The mean viral load was 62411.23 copies/mL (3.8log) in HIV infected children. Plasma concentration of soluble Fas ligands was positively and significantly correlated with the viral load in the sub-group population of HIV infected children (r_s_=0.4; p=0.008) while plasma concentration of soluble Fas receptors negatively and non-significantly correlated with the viral load (r_s_=-0.12; p=0.300). These correlations are summarized in Figure 4 A, Figure 4 B. Taking separately treated and ARV naive children, we observed a negative and significant correlation between the viral load and the Fas L concentration while a positive and significant correlation between the viral load and Fas R was observed with ARV naive children. A positive but not significant correlation was observed between the viral load and FasL concentration in ARV treated children. A negative correlation was observed between the viral load and Fas receptors concentration in the same group. These two correlations are summarized in Table 2.

**Table 2:** Correlation between Fas Ligand (FasL) and Fas Receptor (FasR) plasma levels in HIV infected ART-naives and ART-treated children with HIV-1 Viral Load (VL)

	FasL levels		FasR levels	
	r_s_ p value		r_s_ p value	
VL of HIV infected children	+0.40	0.00	+0.12	0.30
VL of HIV infected ART Naives children	- 0.30	0.04	+0.30	0.04
VL of HIV infected ART Treated children	+0.20	0.03	- 0.26	0.20

r_s_= Spearman’s correlation at 95% confidence interval (CI), VL= Viral load in ART-treated and ART-naives children

## Discussion

The current study aimed at measuring and evaluating the impact of soluble Fas receptors and ligands in HIV/AIDS infected and exposed non-infected children and the correlation with CD4+ cell depletion and HIV-1 RNA load in the sub-group of those infected. Our results showed that the expression level of FasR was higher than that of FasL. Fas L or CD95L is an innate immune factor whose binding with its receptor induces apoptosis. Fas ligand/receptor interactions play an important role in the regulation of the immune system and the progression of HIV infection. Probably there are other ligands that can bind to the Fas receptors because we observed more Fas receptors than Fas ligands. There was an increased levels of Fas receptors and ligands in HIV-1 infected children compared to HIV-1 uninfected children. This has been shown in some previous studies done both on children and adults [13, 14]. These two immune activation markers have been shown to correlate with HIV-1 disease progression in adults and children [15]. Massive CD4+ T cell depletion in acquired immunodeficiency syndrome (AIDS) patients may involve the death Fas receptor, but this role can also be played by other death receptors, such as the TNF and TRAIL receptors, using the same ligand ; explaining while the concentration of ligands was lower than that of receptors [16]. The activity of Fas L and Fas R can explain partly the depletion of CD4 but not fully. This result is contradictory to the findings of Sieg *et al*, 1997, where they showed deficiency of FasL activity on the PBMC surface of HIV infected patients [17].

There was no statistically significant difference in the concentration of FasL between the group of HIV infected children and that of HIV exposed and uninfected children (HEU). It has been shown that HIV upregulates the expression of FasL [18]. In utero, HEU children are exposed to the virus for average 9 month, so as their infected peers. This implies that the immune system of the HEU children is as well activated like that of HIV infected children. In addition, HEU as well as infected infants during their early life, have been exposed to ARV treatment which impacts them negatively by altering their immune system, resulting in activation [19]. They are as well exposed to cotrimoxazole as recommended by WHO, given the increased burden of morbidity and mortality in this specific group of children, particularly from pneumonia [20, 21]. The expression level of FasR in the exposed non-infected children was the lowest compared to control or HIV infected children. It has been shown that peripheral blood lymphocytes expressing FasR were more elevated in HIV-infected individuals than HIV-negative controls [22]. Low expression of FasR in HEU may be due to the elimination of the exposing agent or a bias due to low sample size or to the decrease of this FasR after exposition, transient, observed in early infancy, but no longer present later in life [23]. Further studies will be needed to elucidate on this observation. We showed that the percentage of CD4+ cells and their absolute values were positively and not significantly correlated with the level of both FasR and FasL concentrations in the overall HIV positive children. These results are not concordant with studies of Ikomey *et al* in 2012 [24] where they found a negative correlation with these two markers in two groups of adult populations. On the other hand, the work done by the same authors in 2016 in a group of children showed a negative correlation result with the absolute CD4 value [25], comparable to what was found in the current study. These findings on adults versus infants, just agree with the fact that there is a difference between pediatric and adults immune system and that the immune system of an infant evolve with time until puberty where their immune system is comparable to adult' one.

Unfortunately, we were not able to measure the CD4+ cells count of the HEU children which could have helped do a better analysis by comparing them to those of HIV positive children. A better monitoring of this group of children is crucial and must be considered in national policies guidelines because till now only few longitudinal studies describing the evolution of their immune system over time in Cameroon as these children are exposed in utero to ARVs, HIV antigens and/or other antigens related to other infections during pregnancy had been done [19]. The positive correlation between the level of FasL and the viral load suggest that this biomarker can predict the HIV-1 disease progression, because a higher levels of viral load impacts on CD4+ cells activation by increasing FasL signals which characterized the CD4+ cells death by apoptosis. This significant correlation between FasL and viral load levels corroborate the work done by Ikomey *et al* in 2016 [25] in a population of HIV infected adult in Yaounde where they also noticed that a high level of sFasL depicts a high HIV-1 viral loads and an advanced stage of HIV-1 disease progression.

**Limitations:** the data collected did not allow us to look at the activation' progression over time after HIV exposition in both HIV infected and HEU groups related to the CD4 value and viral load in HIV infected subgroup of children.

## Conclusion

There is a differential expression of FasL and FasR in relation to exposition to HIV virus. We noted a positive correlation between soluble Fas ligand levels and the viral load. Future studies are required to confirm the validity of this correlation, as well as to properly map the evolution of the Fas soluble titres with HIV disease progression in children. This could be a possible solution to monitor HIV infected children in areas where access to routine viral load measurement is problematic. These results indicate that the monitoring of HIV-1 exposed uninfected children and their infected peers must be reinforced in Cameroon as well as in other low income countries. Due to the fact that children and adolescents are somehow different, further longitudinal studies on HEU children and their infected peers from new born to adolescent is highly recommended in order to characterize this growing population in resource-poor settings.

### What is known about this topic

- Previous studies carried out on adults in Cameroon have shown the increase in plasma levels of Fas receptors (FasR) and Fas ligands (FasL) in HIV-positive compared to HIV negative individuals;
- To date, a number of studies have shown evidence of various immunological abnormalities among HEU children (existence of a less effective humoral (antibody, complement) immune response, decreased transplacental transfer of protective maternal antibodies among HEU newborns ; more activated T cell profile as well as a more inflammatory innate immune response in HEU).

### What this study adds

- HEU and HIV infected children express comparable amount of FasL in their plasma.

## Competing interests

The authors declare no competing interests.

## References

[1] Forbes John C, Alimenti Ariane M, Singer Joel, Brophy Jason C, Bitnun Ari, Samson Lindy (2012). A national review of vertical HIV transmission. AIDS.

[2] UNAIDS Global HIV & AIDS statistics-2018 fact sheet.

[3] Nkenfou Nguefeu Céline, Temgoua Saounde Edith, Dambaya Béatrice, Tanguinpundum Charlotte, Colizzi Vittorio, Thèze Jacques (2015). Characterization of Asymptomatic Children Infected with the Human Immunodeficiency Virus at Birth. J AIDS Clin Res.

[4] Newell Marie-Louise, Hoosen Coovadia, Cortina-Borja Marjo, Rollins Nigel, Gaillard Philippe, Dabis Francois (2004). Mortality of infected and uninfected infants born to HIV-infected mothers in Africa: a pooled analysis. Lancet.

[5] Evans Ceri, Prendergast Andrew (2017). Co-trimoxazole for HIV-exposed uninfected infants. The Lancet Global health.

[6] Mofenson Lynne Meryl (2015). Editorial commentary: New challenges in the elimination of pediatric HIV infection, the expanding population of HIV-exposed but uninfected children. Clin Infect Dis.

[7] Dauby Nicolas, Goetghebuer Tessa, Kollmann Tobias R, Levy Jack, Marchant Arnaud (2012). Uninfected but not unaffected: chronic maternal infections during pregnancy, fetal immunity and susceptibility to postnatal infections. Lancet Infect Dis.

[8] Alderson Mark R, Armitage Richard J, Maraskovsky Eugene, Tough Teresa W, Roux Eileen, Schooley Ken, Ramsdell Fred, Lynch David H (1993). Fas transduces activation signals in normal human T lymphocytes. The Journal of experimental medicine.

[9] Eggena Mark Peter, Barugahare Banson, Okello Martin, Mutyala Steven, Jones Norman, Ma Yifei, Kityo Cissy, Mugyenyi Peter, Cao Huyen (2005). T cell activation in HIV-seropositive Ugandans, Differential associations with viral load, CD4+ T cell depletion and coinfection. The Journal of infectious diseases.

[10] Poonia Bhawna, Pauza David, Salvato Maria (2009). Role of the Fas/FasL pathway in HIV or SIV disease. Retrovirology.

[11] Cohen John (1994). Apoptosis: physiologic cell death. J lab Clin Med.

[12] Ikomey George Mondinde, Okomo Assoumou Marie Claire, Atashili Julius, Mesembe Martha, Mukwele B, Lyonga Emilia, Eyoh Agnes, Ndumbe Peter (2012). Plasma concentrations of soluble Fas receptors (Fas) and Fas ligands (FasL) in relation to CD4+ cell counts in HIV-1 positive and negative patients in Yaounde, Cameroon. BMC research notes.

[13] Afran Louise, Garcia Knight M, Nduati Eunice, Urban Britta, Heyderman Robert S, Rowland-Jones Sarah L (2014). HIV- exposed uninfected children: a growing population with vulnerable immune system. Clin Exp Imm.

[14] Sloand Elaine M, Young Neal S, Kumar Princy, Weichold Frank F, Sato Tadatsugu, Maciejewski Jaroslaw P (1997). Role of Fas ligand and receptor in the mechanism of T-cell depletion in acquired immunodeficiency syndrome: effect on CD4+ lymphocyte depletion and human immunodeficiency virus replication. Blood.

[15] Kuhn Klaus, Lotz Martin (2001). Regulation of CD95 (Fas/APO-1)-induced apoptosis in human chondrocytes. Arthritis and rheumatism.

[16] Dianzani Umberto, Bensi Thea, Savarino Andrea, Sametti Selina, Indelicato Manuela, Mesturini Riccardo, Chiocchetti Annalisa (2003). Role of FAS in HIV infection. Current HIV research.

[17] Sieg Scott, Smith Dawn, Yildirim Zafer, Kaplan David (1997). Fas ligand deficiency in HIV disease. Proc Natl Acad Sci USA.

[18] Mitra Debashis, Steiner Melissa, Lynch David H, Staiano-Coico Lisa, Laurence Jeffrey (1996). HIV-1 upregulates Fas ligand in CD'+T cells in vitro and in vivo: association with Fas-mediated apoptosis and modulation by aurintricarboxylic acid. Immunology.

[19] Abu-Raya Bahaa, Kollmann Tobias R, Marchant Arnaud, MacGillivray Duncan M (2016). The Immune System of HIV-Exposed Uninfected Infants. Front Immunol.

[20] Koyi Koyanagi A, Humphrey JH, Ntozini R (2011). Morbidity among human immunodeficiency virus-exposed but uninfected, humanimmunodeficiency virus-infected and human immunodeficiency virus-unexposed infants in Zimbabwe before availability of highly activeantiretroviral therapy. Pediatr Infect Dis J.

[21] Marinda Edmore, Humphrey Jean H, Iliff PeterJ, Mutasa Kuda, Nathoo Kusum J, Piwoz Ellen G (2007). Child mortality according to maternal and infant HIV status in Zimbabwe. Pediatr Infect Dis J.

[22] Gehri Roland, Hahn Sinuhe, Rothen Marilynn, Steuerwald Michael, Nuesch Reto, Erb Peter (1996). The Fas receptor in HIV infection; expression on peripheral blood lymphocytes and role in depletion of T cells. AIDS.

[23] Ruck Candice, Reikie Brian A, Marchant Arnaud, Kollmann Tobias R, Kakkar Fatima (2016). Linking Susceptibility to infectious Diseases to immuneSystem Abnormalities among Hiv exposed Uninfected infants. Frontiers in immunology.

[24] Ikomey Mondinde George, Atashili Julius, Graeme Brendon Jacobs, Mesembe Martha, Eyoh Agnes, Lyonga Emilia, Okomo Assoumou Marie Claire (2016). Fas Mediated (CD95L) Peripheral T-cell Apoptosis Marker in Monotoring HIV-1 Disease Progression in Adults in Yaoundé, Cameroon. Int J Immunol.

[25] Ikomey Mondinde George, Okomo Assoumou Marie Claire, Atashili Julius, Mesembe Martha, Mukwele B, Lyonga Emilia, Eyoh Agnes, Kafando A, Ndumbe Peter (2016). The Potentials of Fas Receptors and Ligands in Monitoring HIV-1 Disease in Children in Yaounde, Cameroon. Journal of the International Association of Providers of AIDS Care.

